# Adaptation of a french e-health tool for suicide prevention in young populations: Modalities and benefits

**DOI:** 10.1192/j.eurpsy.2021.931

**Published:** 2021-08-13

**Authors:** L. Daval, A. Le Jeannic, C. Picot-Ngo, K. Turmaine, K. Chevreul

**Affiliations:** 1 Urc Eco Ile-de-france, AP-HP, Hôtel-Dieu, Paris, France; 2 Eceve - Umr 1123, Inserm, Paris, France; 3 Ufr De Médecine, Université de Paris, Paris, France; 4 Unité D’epidémiologie Clinique, AP-HP, Hôpital Robert Debré, Paris, France

**Keywords:** adolescent, Suicide, e-tool, prevention

## Abstract

**Introduction:**

France’s suicide rate is among the highest in Europe, with the young among the more at risk than others. Several European projects have demonstrated the effectiveness of using e-tools in suicide prevention particularly for hard-to-reach populations. Lessons from StopBlues, an e-health tool (application/website) for suicide prevention in the general population developed in 2018 which was promoted by municipalities and general practitioners, shows the necessity to adapt its content for young people.

**Objectives:**

The objective is to develop an e-health tool, BlueZberry, for suicide prevention targeting adolescents and young adults with psychological pain by adapting StopBlues and its promotional plan.

**Methods:**

The detailed content of BlueZberry and its promotional plan were determined via a literature review and 26 individual and group interviews with experts and youth with StopBlues as a starting part.

**Results:**

The literature review and interviews confirmed the need to adapt the tool according to age of the user since the context and source of psychological pain vary rapidly at this time of life. BlueZberry consists of three modules for age groups 12-14, 15-17 and 18-25 years with specific graphics and messages. Its locally organized promotion should include youth hangouts on top of usual places.

**Conclusions:**

This adaptation of StopBlues will reach a larger audience by offering a more suitable solution for this vulnerable population. A web-portal will serve as an entry point for both StopBlues and BlueZberry where users will be redirected to one of the tools/modules according to their profile and respective needs.

